# PHL9/455: DIKS - The National Diabetes Information and Communication Server

**DOI:** 10.2196/jmir.1.suppl1.e90

**Published:** 1999-09-19

**Authors:** H Sippel, T Baehring, W Scherbaum

**Affiliations:** ^1^Diabetes ForschungsinstitutDüsseldorfGermany

**Keywords:** Health Promotion, Java, Multimedia, Internet, Diabetes

## Abstract

**Introduction:**

Diabetes mellitus has developed to a wide-spread disease. It is known that information about the risks of diabetes could lead to a change in personal behaviour, which lowers the risks of diabetes and diseases caused by it. Esp. early diagnosis could help avoiding diabetes-caused diseases. The National Diabetes Information and Communication Server DIKS shall lead to better information of the public. The Diabetes Research Institute Düsseldorf has started with its development in a project financed by the German Ministry of Health.

**Methods:**

DIKS will cover a wide range of services. As means of presentation are planned: print media, multimedia CDs, touch screen terminals (located in pharmacies, but also in many public places) and of course the Internet. The presentation will be actualized regularly, and attractive enough to guarantee public interest. It is planned to make special presentations for all ages (e.g. children, seniors) and foreign citizens as well (multi-lingual).The Internet offers the possibility for feedback, and profiles of users will be generated. The citizen will have the opportunity to ask questions via Internet. A second level will be the communication between experts. Diabetologist will have a platform for information interchange, publications, and they are asked to participate in the whole project. The DIKS project has just begun. All information will be stored in databases, from where all presentations will be generated dynamically, so that a unique layout will lead to a corporate identity. It is planned to go "online" in about a year.

**Discussion:**

We hope that the DIKS will reach a great part of the public. I should not be just one new web site, which loses its attractiveness over the time.

